# QuantifyMe: An Open-Source Automated Single-Case Experimental Design Platform †

**DOI:** 10.3390/s18041097

**Published:** 2018-04-05

**Authors:** Sara Taylor, Akane Sano, Craig Ferguson, Akshay Mohan, Rosalind W. Picard

**Affiliations:** Affective Computing Group, MIT Media Lab, Massachusetts Institute of Technology, Cambridge, MA 02139, USA; fergusoc@media.mit.edu (C.F.); akshay.mohan@alum.mit.edu (A.M.); picard@media.mit.edu (R.W.P.)

**Keywords:** single-case experimental design, mobile health, wearable sensors, self-experiment, self-tracking

## Abstract

Smartphones and wearable sensors have enabled unprecedented data collection, with many products now providing feedback to users about recommended step counts or sleep durations. However, these recommendations do not provide personalized insights that have been shown to be best suited for a specific individual. A scientific way to find individualized recommendations and causal links is to conduct experiments using single-case experimental design; however, properly designed single-case experiments are not easy to conduct on oneself. We designed, developed, and evaluated a novel platform, QuantifyMe, for novice self-experimenters to conduct proper-methodology single-case self-experiments in an automated and scientific manner using their smartphones. We provide software for the platform that we used (available for free on GitHub), which provides the methodological elements to run many kinds of customized studies. In this work, we evaluate its use with four different kinds of personalized investigations, examining how variables such as sleep duration and regularity, activity, and leisure time affect personal happiness, stress, productivity, and sleep efficiency. We conducted a six-week pilot study (*N* = 13) to evaluate QuantifyMe. We describe the lessons learned developing the platform and recommendations for its improvement, as well as its potential for enabling personalized insights to be scientifically evaluated in many individuals, reducing the high administrative cost for advancing human health and wellbeing.

## 1. Introduction

The rise of mobile devices has ushered in a time of unprecedented data collection through a combination of mobile and wearable sensors as they have become nearly ubiquitous full-time extensions of an individual [1,2]. In fact, nearly two-thirds of Americans are now smartphone owners and more than twenty percent of Americans use wearables, many of which track personal health [3,4]. These data can provide individual users the opportunity to see their habits and patterns quantified [5]. One of the most common ways these data are used is to compare one’s own data with recommended values (i.e., comparing duration of sleep to the recommended eight hours or the number of steps taken to 10,000) and adjust daily behaviors accordingly.

Inherent in this process is a belief that meeting these recommended values will lead to optimum productivity, health, or other such outcomes. However, these recommendations are averages and ideal values may vary significantly in individuals. Although average values are useful, they do not provide the personalized insights that are best suited for an individual to make optimal behavioral choices. For example, an individual may need nine hours of sleep instead of eight to be maximally productive. In some cases, such as “take ten thousand steps daily”, the commonly stated advice is not an official recommendation that has been scientifically validated [6,7]. While many apps and services encourage individuals to meet target behavioral goals, few offer evidence-based and systematic mechanisms to causally connect the effect of behavioral variables such as total sleep duration and physical activity to goals such as productivity, stress and happiness.

Our platform, QuantifyMe, creates a framework that allows people to find their personal optimal behavioral variables (e.g., bed time or physical activity) to achieve their goals (e.g., productivity or happiness) based on evidence-based experimentation. This is done through a single-case experiment design methodology—a methodology that allows for comparison within an individual instead of between groups. In achieving these aims, we are closer to including the general public in dramatically scaling and personalizing the study of daily behaviors.

In this paper, our contribution is three-fold:

- A deeper understanding of the self-experiments that healthy individuals may be interested in, especially healthy, tech-savvy 18–24 years olds
- Development and evaluation (a six-week pilot user study with *N* = 13) of a platform (available at https://github.com/mitmedialab/AffectiveComputingQuantifyMeAndroid) for healthy individuals to conduct self-experiments based on a single-case design methodology (note, since the focus of this paper is how the platform works and how individuals use the platform, we only briefly discuss the individualized results of the single-case self-experiments)
- A collection of insights into participation patterns of healthy individuals when using a single-case based platform designed to help them find their optimal behaviors

The following sections cover a survey of the related work, findings of a study for determining which self-experiments to focus on, and the development and evaluation of the QuantifyMe platform, which we offer free to others to adapt and use.

## 2. Related Work

### 2.1. Quantified Self and Self-Experimentation

Quantified self, also referred to as personal informatics [5] or personal analytics [8], refers to the practice of individuals engaged in self-tracking their biological, physical, or behavioral information [9,10]. Quantified-self applications have progressively moved from being desktop-based to mobile-based systems [3,4,11,12,13]. Current mobile devices and applications can capture behavioral and physiological data such as physical activity [14,15,16,17,18], diet [19,20,21,22], sleep [14,15,23,24,25,26], smoking [27], stress [28,29,30] and social interaction patterns [31] in minimally intrusive ways.

Individuals then use these data to realize a variety of personal goals. Current systems and applications aim to support their users’ goal seeking by providing a mechanism to log data, visualize it, and provide notifications of gaps from target goals. Rooksby [32] mentioned that the key reason for self-tracking is to achieve a certain goal (e.g., walking ten thousand steps) or to determine the relationship between variables. Choe et al. [10] identified similar motivations for engaging in self-tracking behavior:

1. To improve health by monitoring key indicators, identify triggers to specific medical conditions, and determine relationships between variables
2. To improve other areas of life, such as productivity and mindfulness
3. To find new life experiences and learn interesting things about oneself

Thus, self-experimentation to improve health and productivity is a major motivator for self-tracking. However, using these data to understand optimal values of variables and causal relationships in behaviors often lacks scientific rigor [10]. Furthermore, recent research reports that helping users to “interpret, understand, gain motivation and act on their data” is critical to improving user engagement with fitness trackers [33]. Others have found that engagement with fitness trackers is typically in momentary activity instead of long-term behaviors and individuals often face barriers with engaging in long-term data [34,35]. Therefore, an easy-to-use, systematic, automated, and scientifically rigorous approach is needed for self-experimentation.

Recent mobile applications have provided great tools supporting more systematic and personalized self-experimentation [36,37,38,39]. PACO enables individuals to design their own self-experiments for behavior and habit change [36]. Daskalova et al. [37] created SleepCoacher, a mobile app that provides personalized sleep recommendations and tests the effectiveness of these recommendations on sleep quality through guided self-experiments. Daskalova et al. [40] also conducted another study to understand how people run self-experiments and proposed a set of guidelines for running successful self-experiments. Karkar et al. [38,39] developed a mobile application for guided self-experiments to determine the specific foods that may cause symptoms in irritable bowel syndrome (IBS).

Our QuantifyMe platform builds on this work by allowing users to conduct guided self-experiments using a combination of objective sensor data from wearables and self-reports. QuantifyMe also provides self-experimentation across a broader range of variables, and helps users understand the effect of their daily life behaviors (physical activity, sleep duration, bed time, and leisure time) on their goals (happiness, sleep efficiency, stress, and productivity). Using a single-case design methodology, QuantifyMe offers evidence-based, automated and systematic mechanisms for self-experimentation.

### 2.2. Single-Case Experimental Design

Randomized Control Trials (RCTs) are considered a gold standard in determining whether a causal relationship exists between a specific intervention and observed outcome [41]. Traditional RCTs operate across groups with each group receiving a different experimental condition, and are unable to provide individual insights [42]. On the other hand, single-case experimental design (SCED), which was first proposed by Neuringer [43], provides a methodology that allows researchers to evaluate the effectiveness of an intervention on an individual. In the SCED framework, an individual serves as his or her own control and is subjected to different experimental conditions at different time periods [42,44]. Individual differences are especially important to consider for variables such as the optimum level of sleep, physical activity, and leisure time needed, as these may vary according to personality, occupation, and physiology of an individual. Use of a SCED can help overcome the limitations of group-based experiments and can provide personalized insights.

Kratochwill [45] and Barlow et al. [42] provided an overview of single case experimental designs, and recommended four key guidelines for traditional single-case experimental designs:

1. The intervention should be systematically manipulated by the researcher, with defined timing and process.
2. Accepted psychometric measures should be used for agreement between multiple observers.
3. There should be at least three attempts for interventions at different points in time when observing the dependent variable.
4. Each phase should include a minimum of three to five data points.

Following the suggested guidelines, the QuantifyMe platform systematically varies the independent or intervention variable (e.g., bed time) over time for each study participant and then observes its effect on the dependent or outcome variable (e.g., stress level), based on a combination of self-report and sensor data. Based on Guideline (1) above, the algorithmic manipulation of the independent variable is predetermined. In the QuantifyMe platform, the independent variable is varied over four stages, with each stage including at least four data points as suggested in Guideline (4) above. We require multiple consecutive days for each stage to account for lagged differences that commonly occur when measuring mood or adjusting sleeping schedules. However, we do deviate from the guidelines slightly to balance the scientific rigor of experimental design and usability of the platform. For example, we do not presently require multiple observers for any measures (Guideline (2) above), although this is an important recommendation that might also improve participation in the future (e.g., by increasing motivation if you know somebody else is seeing your measurements). Furthermore, each stage is only attempted once in the current design during this initial testing of the platform (in contrast to Guideline (3) above), which was a limit due to the timing available for the original deployment. Nevertheless, the QuantifyMe platform can easily be extended to include repeated attempts for intervention stage. The experimental design is detailed in Section 4.1.

## 3. Survey Study for Understanding Users’ Interest in Self-Experimentation

Before developing the QuantifyMe platform, we conducted an online study to gauge users’ interest in self-experimentation and to find which of 32 self-experiments interested the participants. In particular, participants were asked to rate their interest (i.e., not interested, maybe interested, or interested) in using an app to help them scientifically understand how certain behaviors affect other aspects of their life. For example, participants were asked to rate their interest in conducting an experiment to find out “how the number of steps I take affects my happiness”. On the completion of the survey, they were entered in a lottery to win 1 of 2 $20 gift cards.

The 32 self-experiments were determined by combinations of independent and outcome variables (see Table 1). The possible output variables (happiness, stress, productivity, and sleep efficiency) were chosen from common indicators of wellbeing. The possible independent variables (active time, steps, sleep duration, bed time, meditation duration, outdoor time, fun time, and attending a religious service) were chosen based on the ability to be measured with the Jawbone wearable sensor or because they are relatively easily controllable and actionable [39].

**Figure 1:**
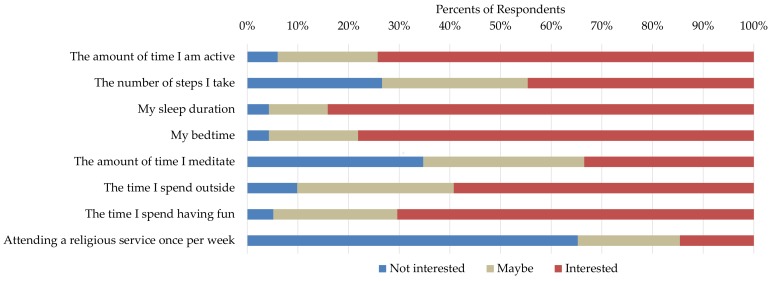
Survey results of self-reported interest in different self-experiments related to productivity. The other outcome measures (happiness, stress, and sleep efficiency) had similar results with sleep duration, bed time was the most popular independent variables, and time spent meditating and attending a religious service were the least popular.

A total of 233 individuals completed the survey (90%: 18–24 years old; 5%: 25–29 years old; and 5%: over 30 years old). We observed similar trends of interest for each of the four outcome variables (see Figure 1 for an example). The most popular independent variable was sleep duration (81–84% of the participants said they are interested), followed by bed time (77–81%), active time (64–74%), and amount of time spent having fun (57–70%). The least popular independent variable was attending a religious event once a week (14–20%), followed by the amount of time meditating (29–33%). The most popular self experimentation was “Does my sleep duration affect my productivity?” (84% interested).

Based on the results of the survey, we decided to focus on four self-experiments—one for each outcome variable:

1. How does my leisure time affect my happiness?
2. How does my activity level affect my sleep efficiency?
3. How does my nightly sleep affect my productivity?
4. How do inconsistent bedtimes affect my stress level?

While we selected these four experiments for their popularity for use in the first version of the app, the QuantifyMe platform was designed to be flexible to accommodate many different kinds of self-experiments, not just these four. This constrained set of self-experiments was chosen to make it easier to evaluate the platform and, more importantly, to give novice self-experimenters a place to start without overloading them with too many options. The specific combinations of outcome variables and independent variables were chosen based on popularity and the likelihood of seeing changes in the outcome when modulating the independent variable. Details about the QuantifyMe platform are described in the next section.

## 4. QuantifyMe Platform for Self-Experimentation

The QuantifyMe platform consists of three parts: a backend Django application, an Android App, and a Jawbone UpMove fitness tracker. The platform could be expanded to other fitness trackers and smartphone platforms. The source code for the platform is available for free here: https://github.com/mitmedialab/AffectiveComputingQuantifyMeAndroid. In the following sections, we describe the QuantifyMe Platform together with a particular study run while using the platform.

### 4.1. Single-Case Experimental Design on QuantifyMe

Typical quantified self experiments measure an outcome (dependent variable) associated with whatever the user’s behavior was at the time, which results in findings that are correlations. In contrast, single-case experimental design tries to mimic a randomized control trial within a person over time; that is, it relies on active (“randomized”) manipulation of an independent variable (e.g., sleep duration) over a period of time and careful observation of a dependent variable (e.g., productivity), all within the same person. This allows the individual user and researchers overseeing a group of single-case self-experimenters to identify possible *causal* relationships between the independent variable and dependent variable.

A traditional suggested design for single-case experiments is an ABAB design, where the *A* phase corresponds to the baseline or control period, and the *B* phase corresponds to the intervention period. This design can be modified as a non-terminated sequential intervention, e.g., $AB_{1}B_{2}B_{3}$, to see the relationship between different magnitudes of the intervention B and their outcomes [46]. This is best suited to our platform, as we are looking to determine the optimum magnitude of the independent variable.

Therefore, we implemented a four-stage design (one baseline stage and three intervention stages) to help users determine optimal behaviors with each stage including 4–7 days of data points as suggested by Barlow and Hersen [42]. In particular, we wanted to include increases and decreases in the behavior to see possible optimal behaviors.

We quantized behaviors into five zones for each experiment (see Table 2). These target behaviors were predetermined by examining common behaviors and correlations between daily behaviors and measures of wellbeing (e.g., how sleep duration affects happiness and stress) based on typical measures found in the SNAPSHOT study, which collected daily behavior, mood, and stress from over 200 young adults [28]. We also included a buffer around each target behavior to accommodate for normal variation and to make it easier for users to achieve the target behavior. This buffer size was also informed by the SNAPSHOT study.

The “randomized” ordering of target goals was chosen as follows. Stage 1 was a baseline measure where users were instructed to maintain their normal behavior. Because a choice needed to be made, we settled on having the middle stage (**O2**) be the last stage for all experiments. We also decided to include at least one increase in the target behavior and one decrease in the behavior. Table 3 lists the different stage order patterns currently used in the QuantifyMe platform. We note that there are other stage patterns that could be used that satisfy these conditions. The platform is flexible to implement any pattern of target behaviors. We note that it might be advisable for researchers to choose the same set of interventions for each individual in a group of self-experimenters to use the hierarchal techniques described by Van den Noortgate and Onghena to generalize results to a larger population [47]. While users of the platform are notified that stages will last for 5–7 days and are notified of their target behavior each day, users in our evaluation study were not made aware of the specific behavioral goal for future stages.

As an example of intervention order, if a user’s average sleep duration during Stage 1 (baseline period) is 6.75 h (i.e., within **O1**), the user would be instructed to sleep 8.5 h, 6.5 h, and 7.5 h during Stages 2, 3, and 4, respectively. However, if the mean of the user’s sleep duration during Stage 1 was 8.75 h (i.e., within **O3**), the user would be instructed to sleep 6.5 h, 8.5 h, and 7.5 h during Stages 2, 3, and 4, respectively. The methodology of imposing behavioral targets adds more structure and validity to determining a causal relationship than simply correlating a behavior with how they feel the next day.

To complete a stage of the self-experiment, we aimed to have 5–7 days where the target behavior was within the target range and the outcome variable is stable as suggested by Kratochwill [45] and Barlow et al. [42] (see also Section 2.2). However, we realized that changing and maintaining specific behaviors would be hard for novice self-experimenter users. Therefore, we developed the following rules for advancing to the next stage or restarting a stage:

1. If the user has (*i*) at least five days within the appropriate target behavior range and (*ii*) a stable output (defined as a self-reported output within 3 points on the Likert scale or sleep efficiencies within 10%, see Section 4.2 for more information), then they are sent to the next stage.
2. If a stage lasts seven days and only four (instead of five) of the days were in the appropriate target behavior range, the user is sent to the next stage. If three or fewer days were in the target range, the stage is restarted. This was done because we need to have several days where the target behavior was achieved, but we do not want the user to be too frustrated with the system.
3. If a stage lasts seven days and the output is unstable, the user is sent to the next stage. This was implemented because we did not want users to become too frustrated with the system restarting when they had high adherence to checking in and following the target behavior.
4. If the user missed checking-in for two days, the stage is restarted. This rule is particularly strict because, if a user did not check-in (report outcomes and receive instructions for the day), he may not know the target behavior for that day.

Put simply, assuming that a user checked in each day, we only count a stage as complete if it has been:

- five days and all days were in the correct target behavior range and the output was stable;
- six days where five of the six days were in the correct target behavior range and the output was stable; or
- seven days where four or five of the days were in the correct target behavior range.

Rule 1, which reflects the best practices for single-case experimental design, requires two conditions to be met. Rules 2 and 3 are implemented as slight deviations to Conditions (*i*) and (*ii*) of Rule 1, respectively, so that we can still maintain the user’s interest throughout the self-experiment. Finally, Rule 4 is required so that users are aware of the target goals. Importantly, these requirements are easily adaptable when using the QuantifyMe platform for other self-experiments if one wants to adjust how strictly individuals must adhere to the best practices of single-case experimental design.

This process is repeated for each of the stages of the experiment until all four stages are completed. After the experiment is complete, the results are analyzed using only the days where the target behavior was in the target range. An example self-experiment can be found in Section 5.4. Therefore, self-experiments will take a minimum of 20 days to complete and use between 16 and 28 days for the data analysis. For example, an experiment will use 16 days for analysis if the participant was only able to maintain in the target behavior for four of the seven days in each stage, while an experiment will use 28 days for analysis if for each of the four stages the output was unstable. Similarly, an experiment will take only 20 days if the participant is able to maintain the target behavior and a stable output for five consecutive days for each of the four stages; however, the experiment may be longer if a participant’s behavior triggers repeated restarting of stages.

### 4.2. QuantifyMe Android App

The Android app was designed with the goal of setting up the self-experiment and letting the user easily “check-in” each day.

When the user first opens the app after installation, it prompts them for demographic data and to self-report their average happiness, stress levels, and subjective sleep quality with seven-point Likert scales (happiness: very unhappy–very happy; stress level: very low–very high; and sleep: terrible–great) and their average total active time per day (0.5, 1, 3, 8, and 12 h). The app also helps the user connect their Jawbone account to our system’s account. Users are then able to browse the self-experiments and select the one they are interested in.

Based on previous mhealth app research [33,48], we speculated that users’ engagement in this app and outcome from the self-experimentation might be related to their self-efficacy and their perceived efficacy of the app. Therefore, after the user selects a self experiment, the user was asked to rate each of the three efficacy questions on a seven-point Likert scale (from 1, Poorly effective, to 7, Highly effective):

1. How effective do you think this app will be in helping you run this experiment? (*App Efficacy*)
2. How effective do you think this experiment will be in getting concrete results? (*Experiment Efficacy*)
3. How effective do you think you will be in carrying out the experiment? (*Self Efficacy*)

Once the efficacy questions are completed, the self-experiment has begun.

Every morning during the experiment, the user is reminded to check-in and fill out a short daily survey. This survey asks about how well they followed the experiment’s instructions (seven-point Likert scale: poor–good), the amount of leisure time they had in the past 24 h, along with happiness, stress, and productivity levels experienced in the past 24 h using seven-point Likert scales (not at all–extremely). Finally, the app reminds the user to sync their Jawbone wearable sensor to Jawbone’s Up App (syncing takes a few seconds). Upon a successful check-in, the QuantifyMe app sends the data to the backend for processing. The backend then responds with both the status of the user’s experiment as well as the daily goal for that day (see Section 4.3 for more information).

**Figure 2:**
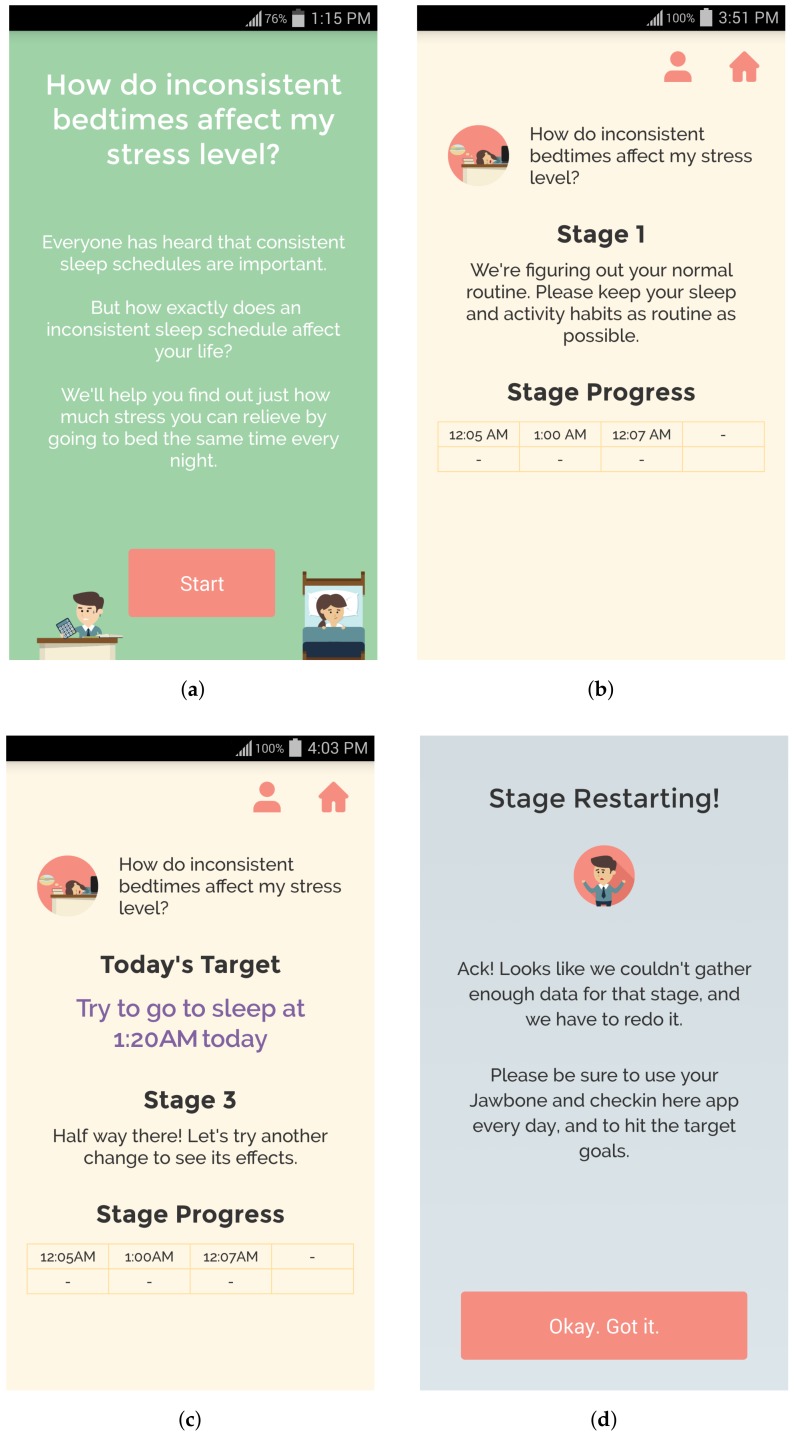
Screenshots of the QuantifyMe Android app: (**a**) information provided before starting an experiment; (**b**) example of instructions during Stage 1 (baseline period); (**c**) example of instructions during Stage 3; and (**d**) example of stage restart notification.

After the user has checked-in for the day, the app presents the user with a screen that lets them view their daily goal and experiment progress during that stage (see Figure 2b,c). In particular, the user can see her recorded behavior for all of the days she has been in that stage. For example, in Figure 2c, we can see that the user has completed three days of Stage 3 and went to bed at 12:05 a.m., 1:00 a.m., and 12:07 a.m. We did not give the user any indication of what the target behavior would be on the next day as we felt that it might bias the results. However, this is something that can be tested to determine the balance between biasing results and providing users with feedback on what to prepare for in the upcoming days.

If a user has failed a stage in the experiment (by not achieving the target behavior range for the targeted number of days, or not providing enough days of data), then they are shown a message prompting them to restart the stage (see Figure 2d). Once an experiment has been completed successfully, the user is shown a success screen with their end results, and the experiment’s results are added to their history, which they can view from the daily goals screen at any time. The user is then again able to select a self-experiment to start (including the one that was just completed).

### 4.3. Backend System

**Figure 3:**
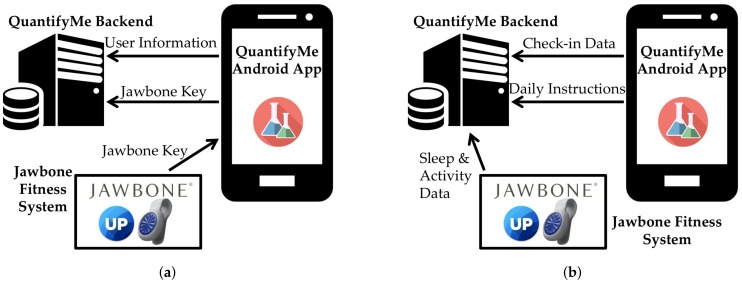
QuantifyMe platform schematics during: (**a**) installation; and (**b**) self-experiment. Note that the Jawbone Fitness System includes the Jawbone backend, the Jawbone Up App, and a Jawbone UpMove tracker.

When a user creates an account in the QuantifyMe app (see Section 4.2), it is sent to the backend (implemented as a Django application) and used for user authentication for all subsequent app requests. The QuantifyMe app also sends an identifier that is used by Jawbone to identify each user, which we associate with our system’s user account (see Figure 3a).

The backend server is set up so Jawbone’s system pushes all updates for all activities to our server automatically as they happen. The server then takes the relevant information involving sleep, activity, and workouts; associates the data with the user with the appropriate Jawbone identifier; and saves it in the database. When the user completes a check-in each day, the data are saved directly into the database. Once the check-in data are saved in the database, the system performs an analysis on the experiment data to determine the instructions for that day (see Figure 3b).

During the initial stage of the experiment, the data are simply gathered without a daily goal to determine the normal base state for the user. Specifically, as discussed in Section 4.1, users are instructed to maintain their normal behavior during Stage 1. When the initial stage is completed, the system determines the daily goals for each of the remaining stages of the experiment (see Section 4.1 for more details on how target zones were determined). In each of the later stages (i.e., Stages 2, 3, and 4), the daily progress is matched against the goal for that stage.

The final results of the experiment are calculated upon the final stage’s completion. The data for every completed stage of the experiment are queried and grouped into days. Then, the system finds the stage that maximizes or minimizes the average of the output measurement, depending on the experiment. The results are then presented to the user through the Android App.

We note that a backend system is used to collect and analyze the data in the QuantifyMe platform so that the researcher helping individuals conduct self experiments could have a central repository for all of the data. This allows researchers to adapt the analysis, if necessary, in an agile way (i.e., without having to have all users install a new version of the app). Furthermore, we wanted to make sure that if the user accidentally deleted his/her app or data that we could still use this data for analysis and reset the individual to the appropriate place in the self-experiment.

## 5. App Evaluation Study Results

We conducted a six-week pilot study to evaluate the QuantifyMe application with 13 participants (four males and nine females; age: 18–27). Participants were recruited through the campus mailing lists and needed to be Android phone users. During an information session, participants were informed of the study protocol and interested individuals gave written informed consent to participate. The protocol was approved by MIT’s Institutional Review Board.

All participants filled out a pre-Study survey which included the Perceived Stress Scale-10 [49] and the Big Five Personality Inventory [50]. The pre-study survey was administered because we are interested in how individuals with different personalities and stress levels interact with the QuantifyMe platform.

Participants then chose a self-experiment they liked, which was continued for six weeks. Participants were allowed to choose their own self-experiment to increase their motivation to complete it. If a participant completed a self-experiment, then they were asked to choose another one to participate in. If the participants stopped using the app or syncing their wearable sensor data for a few days, a study investigator contacted the participants to determine if they had a problem using the app or the wearable sensor and encouraged them to continue participating by checking-in to the QuantifyMe App and syncing their Jawbone.

At the end of the study, the participants filled out a post-study survey including a System Usability Scale [51] and questions about their experience in the study, including their favorite and least favorite parts of the app, what they would have changed about the app, what was difficult to understand in the app, and how they felt about finishing the study. Participants were also prompted to give any other feedback they had through an open-ended question.

Participants were compensated with a $20 gift card upon completion of the six-week study (i.e., filling out a pre- and post-study survey) regardless of whether or not they were able to complete a self-experiment. The compensation was deliberately kept low to try to avoid motivating the participants extrinsically with money.

The following subsections discuss the results of the QuantifyMe App Evaluation Study. The main findings are summarized as follows:

- Participants have lower average Perceived Stress Scale (PSS) scores than are typical in their age group.
- Participants self-reported neutral happiness, stress, and sleep quality before starting the study.
- Participants have lower than average extraversion, conscientiousness, neuroticism, and openness.
- Participants have higher than average agreeableness.
- Choice of self experiment matches the distribution of the self experiments in the survey study discussed in Section 3.
- Participants reported neutral efficacy scores.
- Some correlations were found between efficacy and personality types (agreeableness and conscientiousness).
- Participants checked-in to the app on 75.6% of the days but they adapted their behaviors to the target ranges for 22.5% of the days.
- Self-reported adherence to the target behaviors was significantly higher than objective adherence to target behaviors.
- Adherence rates are correlated with personality types (agreeableness and conscientiousness).
- Check-in adherence and objective adherence to the target behaviors were highly correlated with self-reported efficacy.
- Participants rated the QuantifyMe Android app as slightly above average on the System Usability scale.

### 5.1. Pre-Study Surveys

The average pre-study Perceived Stress Scale (PSS) score of the study participants was 12.5 with a standard deviation of 5.0, which is lower than the average in the age group 18–29 (mean: 14.1; and SD: 6.2) (note: PSS scores range from 0 to 40, with higher scores being more stressed) [49]. That is, our participants, on average, initially reported being less stressed than others in their age group.

According to their self-reports, the participants were neutral (i.e., scores of approximately 4 on a Likert Scale from 1 to 7) in happiness, stress level, and sleep quality (see Table 4). Our participants had lower extraversion, conscientiousness, neuroticism, and openness and slightly higher agreeableness than the mean values in the same age group (see Table 5 for mean values for our study and Srivastava et al. for mean values for the same age group [52]). However, due to the small sample size, we do not test for significant differences in the Big Five Personality Traits compared to the general population.

### 5.2. Self-Experiment Selection

Among the four experiments on the app, five people chose “effect of sleep duration on productivity,” four people chose “effect of leisure on happiness,” two people selected “effect of sleep variability on stress,” and two selected “effect of steps on sleep efficiency”. This distribution matches that of the survey we conducted before designing QuantifyMe (see Section 3).

### 5.3. Efficacy

All of the efficacy scores from participants averaged close to neutral (see Table 6). This means our participants did not fully expect that the app would be effective in helping them run the self experiment (app efficacy) or providing concrete results (experiment efficacy). A neutral score on the self-efficacy question implies that participants were not confident that they could complete the study, even though they were interested in trying. We also found that app efficacy was correlated with experiment efficacy (r=0.6, p<0.05, Spearman Correlation).

In addition, we examined if the efficacy scores were related to the participant’s personality types. We found higher agreeableness was related to higher app efficacy (r=0.65, p<0.05) and experiment efficacy (r=0.72, p<0.01) as expected since individuals that are more agreeable have a tendency to be more trusting [50]. Higher conscientiousness was related to lower self efficacy (r=−0.59, p<0.05). This finding was surprising, as individuals that score high for conscientiousness are often described as self-disciplined and achievement striving [50]; however, higher conscientious individuals may have a better understanding of the upcoming demands in their lives and were therefore better able to evaluate how easy it would be for them to complete the self-experiment.

We also analyzed how participants’ personality types affect the app usage; we suggest potential improvements to increase efficacy and engagement in Section 5.6.

### 5.4. Self-Experiment Adherence

During the six-week study, three participants dropped out for various reasons including phone malfunction, side-effects of an unrelated medication, and the self-experiment (inconsistent bedtimes and stress) being too difficult to complete. Thus, 10 participants completed the study (i.e., used the QuantifyMe app for six weeks); however, only one participant successfully completed all parts of a full four-stage scientific self-experiment during the six-week study. The participant that completed a self-experiment chose the self-experiment “effect of duration of leisure time on happiness.” For this individual, there was no effect observed.

**Figure 4:**
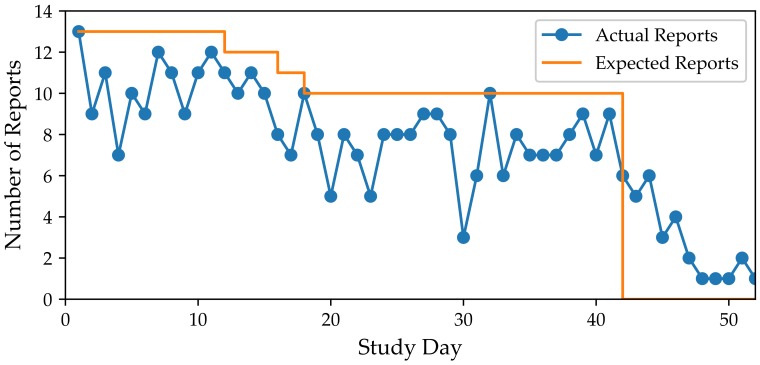
The number of participants that checked-in to the QuantifyMe App on each day of the study. The orange line displays expected number of reports for each day.

The average adherence rate for checking-in (i.e., *check-in adherence*) to the QuantifyMe app was 75.8%. Figure 4 shows the number of reports for each day in the study. We note that *check-in adherence* remained stable throughout the study, and decreased rapidly after the study ended. *Check-in adherence* rates varied widely with four participants checking-in on less than 65% of the days and three participants checking-in on more than 90% of the study days.

Participants also reported their adherence to the target behaviors (i.e., *self-reported adherence*) on a Likert-scale from 1 (did not follow instructions) to 7 (followed instructions exactly) each time they checked-in. Participants self-reported a median adherence of 4; however, this only includes participants that actually checked-in and does not account for those who did not.

**Figure 5:**
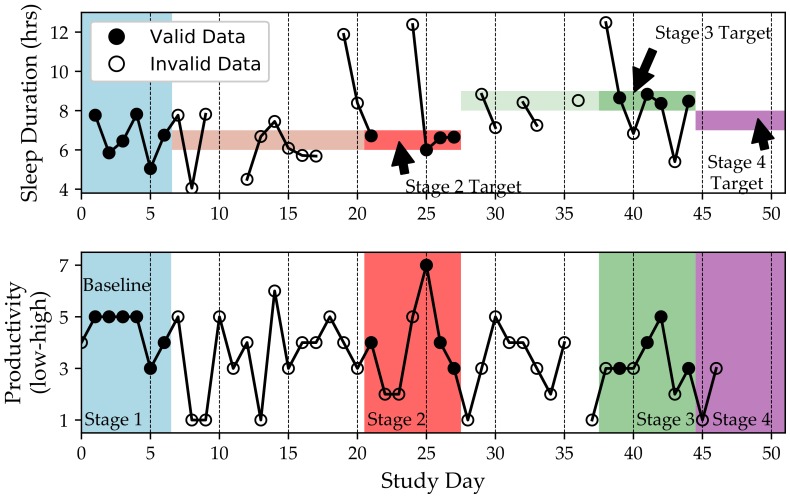
Example of a participant’s self-experiment. The (**top panel**) shows filled circles where the independent variable, sleep duration, was reported within the study range requirements, and empty circles where it was reported as outside the required range. The (**bottom panel**) shows all reports for the outcome variable: self-reported productivity. The shaded areas (blue, red, green, and purple) mark the four stages of the study, only the first three of which met the requirement of “at least four days” of data (darkened circles). In the top panel, we can see that these shaded areas also display the bounds of target behavior (except for the baseline stage, which had no target behavior and therefore no bounds). Only valid data are used in analysis.

In comparison, we found that, on average, participants adapted their behaviors to be in the target range for only 22.5% of the study days (i.e., *objective adherence*). This lack of adherence to self-experiment instructions was the main reason why only one participant completed a self-experiment in the six-week period. In other words, participants had trouble adjusting their behaviors to match the self-experiment instructions (see Figure 5 for an example). This resulted in many stage restarts because we required participants to check-in at least five out of seven days and be within the target behavior range for at least four of the seven days.

While this adherence requirement may seem onerous (and typically is not followed by most quantified-selfers), it is important scientifically for helping reduce the effects of confounders that might causally influence both “the input behavior” and “the outcome”. Requiring adherence when the person may “not feel like adhering” helps test if the behavior really makes a causal difference on the outcome.

We did not find any statistically significant relationships between *self-reported adherence* and *objective adherence*. However, we did observe a proportional relationship between *check-in adherence* and *objective adherence* (r=0.44), but the correlation coefficient was not statistically significant. This finding re-enforces the need to have objective measures as relying on a participant’s memory does not accurately reflect the participant’s actual adherence. This also implies that if we could improve our app to increase *objective adherence*, then users would be able to complete more self-experiments.

We also analyzed whether experiment types influence *self-reported adherence* and *objective adherence*. We compared them between the top two experiments “effect of sleep duration on productivity” (*N* = 5) and “effect of leisure on happiness” (*N* = 4); however, no differences were found (p>0.5).

Figure 5 shows an example of a participant’s self-experiment. We note that the participant is checking-in to the QuantifyMe app nearly every day (as evidenced by the productivity scores reported on 46 out of 47 days). However, we have reports of sleep duration for only 34 of the days. Furthermore, the participant does not keep her sleep duration in the target range (denoted by the vertical span of the colored bars in the top panel) for 25 of the 46 days of the self-experiment. For example, study days 7–20 were attempts to complete Stage 2, but the participant failed to complete the stage due to not achieving the target sleep duration behavior (shown in light red). We also note that the last stage (purple) was only started and never finished. The Jawbone UpMove did not automatically detect sleep episodes, but other wearable devices do log sleep; future studies should be conducted with wearables that automatically log sleep vs. wake to alleviate the problem of requiring the participant to log this regularly.

The results of the self-experiment shown in Figure 5 would be computed as follows. Productivity scores on Days 1–6 would be averaged and form the baseline productivity. The productivity scores on Days 21, 25, 26, and 27 (days when the participant achieved the target sleep duration: 6.5 h) would be averaged and form the Stage 2 productivity score (i.e., productivity when sleep duration is 6.5 h) since during those days the participant was able to adjust their sleep behavior to the target goal of 6.5 h of sleep. Similarly, Days 39, 41, 42, and 44 would be averaged to form the Stage 3 productivity score (i.e., productivity when sleep duration is 8.5 h) and, if Stage 4 were completed, the days where the sleep duration was in the target range would be used to compute the average productivity for 7.5 h of sleep. These four scores (baseline and Stages 2–4) would then be compared to determine the highest productivity. The corresponding sleep duration would be reported to the participant as their personalized optimal sleep duration for achieving high productivity.

### 5.5. Post-Study Survey

**Figure 6:**
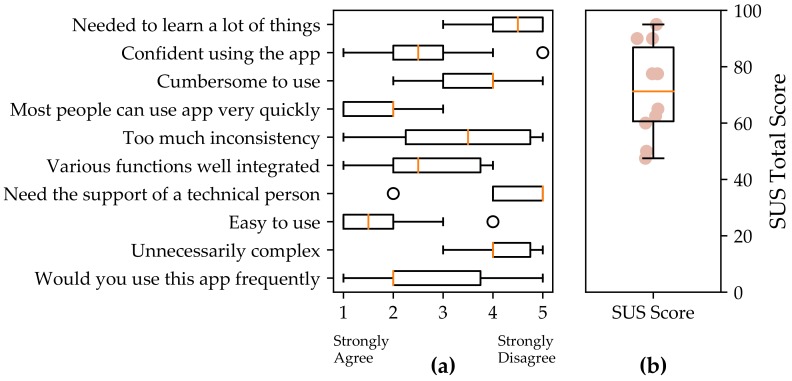
Results of the System Usability Scale: (**a**) the distribution of responses for each question; and (**b**) the distribution of the SUS total score.

The System Usability Scale (SUS) results presented are from 10 study participants and excludes three who had technical difficulties with use of the app on their phone (which were resolved with an updated version of the app). The SUS for the QuantifyMe app had a mean of 71 (out of 100) with a standard deviation of 17 (Figure 6). A SUS score of 68 is considered average [53]. Therefore, the app scored slightly above average for usability.

Post-study free-form comments were collected and analyzed from all 13 participants. Seven out of 13 study participants indicated that the daily survey and stage progress allowed them to be more aware of their behavioral patterns and prompted introspection of their lifestyle. The participants in the study appreciated the morning notifications and visualizations of the monitored data including physical activity and sleep data, which is consistent with what users find useful in available health apps. They also liked the simple interface design. In addition, one participant also specifically mentioned that it was useful to have the application guide her through the experiment, and appreciated not needing to do any calculations.

Most study participants indicated their disappointment in not being able to complete the full four-stage self-experiment during the six-week study. One participant said, “It was hard to follow the instructions about sleep schedule because it was more than I am used to and I wasn’t able to plan my schedule around that”. Another participant said, “Worrying about when I wasn’t able to complete the app instructions” was their least favorite aspect of the study. These comments were consistent with the behavioral lack of success in changing the independent variable in the various studies.

### 5.6. Relationships among Adherence, Personality, Efficacy, and Usability

Because adherence was an important factor to individual participant’s success in completing a self-experiment, we looked into the relationships between adherence and personality, efficacy, and usability. We found that *check-in adherence* was statistically significantly associated with agreeableness (r=0.93, p<0.01) and *objective adherence* was significantly related with agreeableness and contentiousness (r=0.78, p<0.01 and r=0.67, p<0.05, respectively). This finding agrees with previous studies, which have shown that adherence to clinical treatment (e.g., rehabilitation and, medication) is related to personality types [54,55].

We also analyzed adherence and pre-study self-reported efficacy. We found that *check-in adherence* and self-reported efficacy are highly correlated (app efficacy: r=0.65, p<0.05; experiment efficacy: r=0.86, p<0.01, and self efficacy: r=0.90, p<0.01). We also found that *objective adherence* is statistically significantly associated with experiment efficacy and self efficacy (r=0.70, p<0.01 and r=0.74, p<0.01 respectively). Finally, we found that higher app efficacy was related to higher *self-reported adherence* (r=0.70, p<0.01); however, app efficacy was not related to *objective adherence*. This means that those users who perceived that the app was likely to be useful before the study started had a higher rate of self-reported instruction adherence, but did not necessarily do a better job of adhering to the experiment instructions.

In addition, we found that a higher SUS score was related to higher experiment efficacy (r=0.67, p<0.05), but we could not find any statistically significant relationships between SUS scores and *self-reported adherence*, *objective adherence* or *check-in adherence*. This means usability scores were related more to a user’s perceived efficacy of the experiment, rather than to their actual behaviors in the self-experiment.

## 6. Discussion

### 6.1. Usability of the QuantifyMe Platform

As discussed in Section 5.5, the participants of the evaluation study rated the QuantifyMe Android app as slightly above average for usability. However, the behavior required by the single-case experiment design was much harder for participants to adhere to than we expected, as only one individual completed a four-stage self-experiment during the six-week evaluation study. In the post-study survey, several participants expressed that some of the experiments were too difficult to complete. Going forward, a researcher using the QuantifyMe platform may wish to choose study goals to better match the abilities of the individuals they are studying, or provide more support for participants to meet the goals demanded by the independent variable. Furthermore, although participants had a hard time adapting their behaviors to the target goals, the QuantifyMe platform itself made it easier for the researchers overseeing the single-case experiments because it took care of tracking behavior and notifying individuals when they needed to restart a stage. In this way, the QuantifyMe platform makes it easier to conduct single-case experiments.

In the following sections, we will further explore how the QuantifyMe platform could be improved and adapted for other research contexts. These suggestions can also be extended to be applicable to other applications that seek to help users engage with their data.

### 6.2. Data Visualizations

Four out of 13 study participants suggested having additional visualizations that would allow them to see the history of their objective data from the Jawbone UpMove and daily check-ins. In our QuantifyMe app, we did not supply any visualizations of the data until they completed the study because showing the participant the results could bias their behaviors and responses. However, we did not prevent the participants from viewing the visualizations available in the Jawbone Up App. The desire for additional visualizations further validates that users are interested in personalized insights and their behavioral patterns to better manage their lifestyle. Future platforms might specifically consider the trade-off between showing mid-experiment results and biasing results.

### 6.3. Enhancing Application Usability by Increasing Transparency

Users suggested that having explanations of the data collection process for each of the variables would be useful, especially for sleep data. One of the interesting usability issues arose due to the unusual time of some of our participants going to bed past midnight in a given day, and thus the day the sleep counted for was confusing to participants. As one study participant mentioned: “It was difficult to understand how the app made its measurements and what was considered a night. As a college student, sometimes I wouldn’t go to bed until 7 am and wake up at 3 pm, but I never knew if the app was picking up on that”. This issue was mentioned by four out of 13 study participants.

Explaining the background of the self-experimentation and the scientific evidence in detail during the study would be helpful to improve efficacy. As one study participant mentioned “I had a lot of information from the info session, but maybe it would have been nice to have some of the mechanics explained at the beginning (of the app)”. In addition, different strategies to engage participants in the system could be designed based on their personality types [56].

The results from these pilot studies are valuable to further improve the design of self-experimentation systems. Based on our results, we recommend that future versions of the QuantifyMe platform and similar platforms need:

1. An adequate explanation or motivation of scientific single-case experiment design to be provided throughout the use of the system—so adherence to a behavior is held for a sufficient number of days within each stage in order to properly evaluate its effects.
2. Mechanisms that support such adherence, repeatedly, even as demands change in the participants’ life.

While the latter mechanisms are a part of most long-term behavior change programs, this study underscored that even short-term periods of behavior change, as part of scientific quantified-self studies, may require significant support for users (e.g., providing encouragement to continue with the intervention or reminders to complete self-reports). Further, the support needed may be of a slightly different nature: For example, while classic behavior support mechanisms may help you design time in your schedule to sleep longer, e.g., to achieve the “sleep between 8 and 9 hours per night four of the next seven nights”, it can require a different kind of motivation to make yourself sleep shorter when you are really tired and your schedule would permit you to sleep in, but your app says you need to sleep between 6 and 7 hours per night for at least 4 nights.

### 6.4. Limitations

This work has several limitations. We assumed that self-experimenters would have enough motivation to maintain the different behaviors being tested during each study stage. Clearly, better mechanisms to encourage behavioral compliance are needed. In the future, we suggest that similar platforms incorporate external motivators to help novice self-experimenters. The study participants did not have familiarity with single-case experimental design, and an information session at the beginning of the study was reported to be insufficient.

Participants suggested it would be valuable to have additional information and motivation about the single-case design methodology such as the number of stages and notifications of why a particular experimental stage would need to be restarted if they did not reach the target for the independent variable. In the future, we recommend that providing study participants with the upcoming targets multiple days ahead and clarifying what flexibility is and is not going to be a set back in the study may allow them to plan for the interventions in advance and increase adherence. While this information (approximate length of stages and target behavior zones) was presented to all participants at the beginning of the six-week study, future platforms ought to include this information throughout the self-experiment to help novice self-experimenters understand the scientific process.

Our population was a set of busy, healthy individuals who think they may be interested in trying to find optimal behaviors; as such, they are not likely to be as motivated to try new behaviors as an unhealthy population who seeks treatment. We also designed multiple targets participants had to meet (instead of sticking with one intervention), adding additional challenges to engaging our healthy population.

Because most participants in our studies were university students who are savvy about technology, experimental design and statistics, these results might not generalize to other populations. In addition, the participants may have been fairly homogeneous in terms of experiments they may be interested in. One sign supporting this was that the most popular experiment selected was related to the amount of sleep needed. However, all of the participants in the pilot study were novice self-experimenters, which suggests that other tech-savvy novice self-experimenters may encounter similar challenges.

Finally, throughout this study, we were reminded of the complexities of modeling human behavior and experiences during normal daily behavior. For example, in the course of the six-week study, some of our participants had three holidays from school, daylight savings time ended, and a major US election occurred. Additionally, these participants had to keep up with school and employment demands. However, we believe that single-case experimental design, implemented with a platform that tracks regular input data, is better suited to account for the complexities of life than a randomized controlled trial because of the ability to obtain frequent and regular time-stamped reports of (compliant or not) behavior, adapt the study sequentially (e.g., restarting if needed), and account for anomalies in the data (e.g., not diminishing individual effects via group averages).

## 7. Conclusions

In this work, we design, develop, and evaluate a novel platform for users to conduct single case self-experiments in a scientific and automated manner. The QuantifyMe platform is designed to create a framework for novice self-experimenters to find their personal optimal behavioral variables to achieve their goals by automating the single-case experimental design process within a mobile application. We designed our platform to allow individuals to chose from four single-case design experiments (chosen to based on results from an interest study) to help them build a model of how variables such as sleep, activity, or leisure time affect their happiness, stress, productivity, and sleep. Although participants in the pilot study are constrained to choose between four experiments, the QuantifyMe platform itself is not constrained to these experiments. In fact, it can include additional independent and dependent variables. The platform is provided in open-source for other researchers to modify for their specific interests (see https://github.com/mitmedialab/AffectiveComputingQuantifyMeAndroid).

In a pilot study, we find that, although target-behavior compliance for the independent variable was low across the four stages, our participants still expressed interest in having such a system to determine personalized optimal behaviors. Future versions of the QuantifyMe platform should include methods of increasing compliance via maintaining motivation on a daily basis and better preparing participants to be able to hit the target behaviors.

In the future, we hope that a platform like QuantifyMe will be able to automatically administer single case experiments to many individuals without the high administrative cost that is currently associated with single-case experimental design; thus, we hope the contributions of this work will help advance human health and wellbeing by providing science-based personalized insights.

## Figures and Tables

**Table 1:** Independent and outcome variables used for the interest study.

Independent Variables	Outcome Variables
the amount of time I am active	happiness
the number of steps I take	stress
my sleep duration	productivity
my bedtime	sleep efficiency ^1^
the amount of time I meditate	
the time I spend outside	
the time I spend having fun	
attending a religious service once per week	

^1^ Computed as minutes asleep/minutes spent in bed.

**Table 2:** Definitions of target zones of behaviors for each experiment.

	Zone Goal					Target Zone Size
	Under	O1	O2	O3	Over	
**Number of Steps (#)**	<6500	8000	11,000	14,000	>15,500	±1500
**Bed Time Variability (±min)**	<15	30	60	90	>105	±15
**Sleep Duration (h)**	<6	6.5	7.5	8.5	>9	±0.5
**Leisure Time (min)**	<15	30	60	90	>105	±15

**Table 3:** Stage order is determined by the user’s behavior during Stage 1.

Stage 1 (Baseline)	Stage 2	Stage 3	Stage 4
O1	O3	O1	O2
O2	O3	O1	O2
O3	O1	O3	O2
Under	O1	O3	O2
Over	O3	O1	O2

**Table 4:** Pre-study self-reported measures (Likert Scale: 1 (bad) to 7 (excellent)).

	Happiness	Stress	Sleep Quality
Mean	4.1	3.5	3.6
St. Dev.	1.7	1.7	1.0

**Table 5:** Pre-study Big-Five personality scores (1 (low) to 5 (high)).

	Extraversion	Agreeableness	Conscientiousness	Neuroticism	Openness
Mean	2.6	3.7	3.2	3.1	3.7
St. Dev.	1.0	0.7	0.7	0.9	0.6

**Table 6:** Efficacy results (1 (poorly effective) to 7 (highly effective)).

	App Efficacy	Experiment Efficacy	Self-Efficacy
Mean	3.7	4.2	3.9
Median	4	4	4
St. Dev.	1.1	1.6	1.0
